# 720. Efficacy of Nifurtimox + Eflornithine in the Treatment of African Trypanosomiasis. Systematic Review

**DOI:** 10.1093/ofid/ofab466.917

**Published:** 2021-12-04

**Authors:** Jessica Hidalgo, Raghavendra Tirupathi, Juan Fernando Ortiz, Stephanie P Fabara, Dinesh Reddy, Ali A Rabaan, Jaffar A Al-Tawfiq

**Affiliations:** 1 Universidad San Francisco de Quito, Quito, Pichincha, Ecuador; 2 WellSpan Health, Chambersburg, Pennsylvania; 3 Larkin Community Hospital, Miami, Florida; 4 Universidad Catolica De Santiago De Guayaquil, Guayaquil, Guayas, Ecuador; 5 Rajiv Gandhi University of Health Science, Hyderabad, Telangana, India; 6 Johns Hopkins Aramco Health Care, Dhahran, Al Bahah, Saudi Arabia; 7 Johns Hopkins school of medicine, Dhahran, Al Bahah, Saudi Arabia

## Abstract

**Background:**

Sleeping sickness is an infectious disease transmitted mainly by the Trypanosoma Brucei, with the tsetse fly as a vector. The condition has two stages: The hemolymphatic and the meningo-encephalitic stage. The second stage is caused mainly by the Trypanosoma Brucei Gambiense. The treatment of the second stage has changed from melarsoprol, eflornithine, to now nifurtimox-eflornithine (NECT). This systematic review will focus on the efficacy and the toxicity of the medication.

**Methods:**

We use PRISMA and MOOSE protocol for this review. On figure 1, we detail the methodology used for the extraction of information from the systematic review. To assess the study's bias, we used Cochrane Collaboration’s tool for risk assessment of the clinical trials and the Robins I tool for the observational studies.

**Results:**

We collected four clinical trials and two observational studies after an extensive search. Three clinical trials showed that NECT was non-inferior to eflornithine with the following cure rates (NECT VS eflornithine): 1) 96.3% vs. 94.1% ; 2) 90.9% vs. 88.9%; 3) 91.6% vs. 96.5%. An additional clinical trial revealed that the proportion of patient discharge from the hospital was 98.4% (619/629); 95% CI [97.1%; 99.1%]). The two observational studies discussed the pharmacovigilance of the drug and toxicity related to NECT. In one study, patients treated with NECT, 589 (86%) experienced at least one adverse effect (AE) during treatment, and 70 (10.2%) experience serious AE. On average, children experienced fewer AEs than adults. In the other study at least one AE was described in 1043 patients (60.1%), and Serious AE was reported in 19 patients (1.1% of treated), leading to nine deaths (case fatality rate of 0.5%). The major limitations of the studies were the lack of blinding because most of them were open-label. Also, there was heterogenicity in the definition of the outcomes in the observational studies.

PRISMA Flow Chart

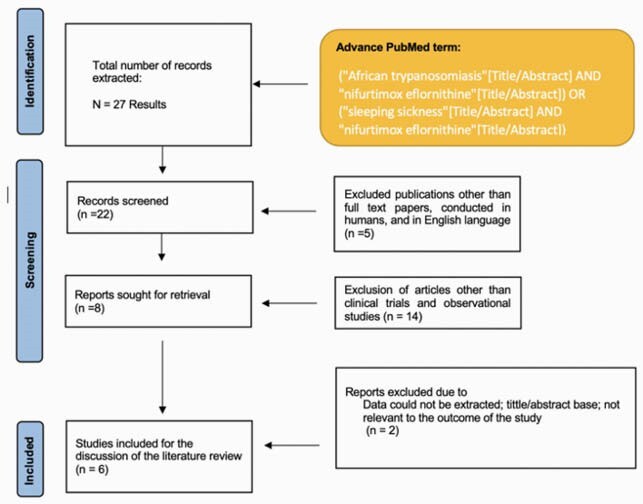

**Conclusion:**

NECT is not inferior to eflornithine, and the proportion of patients discharged from the hospital alive showed favorable results. The observational studies revealed a high frequency of AE. However, NECT is more convenient and safe than Eflornithine and Melarsoprol.

**Disclosures:**

**All Authors**: No reported disclosures

